# Connectivity between the seizure onset zone and the thalamus correlates with seizure outcomes in thalamic responsive neurostimulation

**DOI:** 10.1002/epi.70052

**Published:** 2025-12-19

**Authors:** Varun R. Subramaniam, Andy Ho Wing Chan, Lara Marcuse, Madeleine Fields, Maite La Vega‐Talbott, Daniel D. Cummins, Juan A. Barcia, Hesham T. Ghonim, Lakshman Arcot Jayagopal, Yunju Im, Saadi Ghatan, Fedor Panov, Josue M. Avecillas‐Chasin

**Affiliations:** ^1^ Department of Neurosurgery Icahn School of Medicine at Mount Sinai New York New York USA; ^2^ Department of Neurology Icahn School of Medicine at Mount Sinai New York New York USA; ^3^ Department of Psychiatry Icahn School of Medicine at Mount Sinai New York New York USA; ^4^ Department of Neurosurgery Hospital Clinico San Carlos Madrid Spain; ^5^ Department of Neurology University of Nebraska Medical Center Omaha Nebraska USA; ^6^ Department of Biostatistics University of Nebraska Medical Center Omaha Nebraska USA; ^7^ Department of Neurosurgery University of Nebraska Medical Center Omaha Nebraska USA

**Keywords:** corticothalamic, responsive neurostimulation, thalamocortical, tractography, white matter

## Abstract

**Objective:**

Thalamic responsive neurostimulation (RNS) is a surgical option for patients with drug‐refractory epilepsy. However, it is unclear whether thalamic connectivity with the seizure onset zone (SOZ) has a role in clinical outcomes. Here, we aim to investigate the clinical utility of the connectivity between the SOZ and the thalamus for thalamic RNS targeting.

**Methods:**

Retrospective analysis was made of 12 patients treated with thalamic RNS. Clinical features and Engel scores were recorded. Patients were divided into responders, partial responders, and nonresponders based on seizure frequency reduction at last follow‐up. Structural connectivity between the SOZ and the whole thalamus was calculated using patient‐specific tractography. RNS electrodes were used to model the volume of tissue activated (VTA) with stimulation parameters at last follow‐up based on individualized electrode locations. The patient's VTAs were then used to identify thalamic areas with high or low probability of connectivity with the SOZ and how they were associated and correlated with clinical outcomes using nonparametric Mann–Whitney *U* and Spearman correlation tests.

**Results:**

Seven patients were responders, three nonresponders, and two partial responders. Thalamic nuclei targeted included anterior nucleus of thalamus and centromedian nucleus. Cortical areas of the SOZs included medial prefrontal, supplementary motor, cingulate, orbitofrontal, insular, mesial temporal, and lateral temporal cortices. Stimulation of thalamic areas with higher connectivity between the SOZ and the thalamus was associated with a clinical response of >50% reduction in seizures (*p* = .017). Furthermore, higher degree of tract activation between the SOZ and the thalamus was correlated with better seizure outcomes at last follow‐up (*r* = .78, *p* = .004).

**Significance:**

Greater recruitment of white matter connections between the SOZ and thalamus is associated with clinical response and may correlate with improved seizure outcomes during thalamic RNS. Using tractography to map the patient‐specific “thalamic seizure network” and the surgical targeting of these connections may result in improved clinical outcomes in patients treated with thalamic RNS.

**Key points**

- The connections between the cortical areas harboring the SOZ and the thalamus show a complex gradient of connectivity along several thalamic nuclei.
- Stimulation of these connections between the SOZ and the thalamus is associated with clinical response and positively correlated with seizure outcomes.
- Findings support the idea that targeting the “thalamic seizure network” may improve outcomes in neuromodulation for epilepsy, moving away from traditional nuclei‐based targeting to a more personalized network‐based approach.
- Patient‐specific tractography can be used to map the connections between the SOZ and the thalamus to prospectively target those connections.

## 1 INTRODUCTION

Responsive neurostimulation (RNS) is a US Food and Drug Administration‐approved surgical option for patients with drug‐resistant epilepsy (DRE) who are not eligible or do not respond to resection or ablation. RNS is delivered either at the seizure foci (if known) or at targets that are believed to be part of the seizure network. It is well known that epilepsy is a network disorder,^1^ and therefore the identification of the seizure network is crucial for improved clinical outcomes. The thalamus is one of the targets for RNS in the treatment of DRE due to its wide connections with cortical areas and therefore a critical part in the seizure network given its involvement in seizure propagation and generalization.^2^ However, selecting the specific area to stimulate in the thalamus remains uncertain in most patients with focal and multifocal DRE. Traditionally, the thalamus has been targeted with deep brain stimulation (DBS) to treat different epilepsy syndromes in which there is no information about the seizure onset zone (SOZ). However, the same targeting principles of thalamic DBS are being applied to RNS despite the potential variability in the seizure networks between patients. The traditional thalamic targets include anterior nucleus of the thalamus (ANT) and centromedian nucleus (CM). From the network standpoint, ANT has been used for patients with limbic/temporal lobe epilepsy and the CM for frontal/parietal lobe (sensorimotor) epilepsy and Lennox–Gastaut syndrome.^3^, ^4^ Other thalamic nuclei have been proposed as targets for neuromodulation including the pulvinar (Pulv)^5^ and mediodorsal nucleus (MD) based on the theoretical connectivity between these nuclei and cortical areas in the parieto‐occipital and prefrontal cortex, respectively.^6^ Despite this, the current selection of thalamic targets for RNS does not involve patient‐specific connectivity information between the SOZ and the thalamus. Tractography is an imaging technique that calculates structural connectivity in a noninvasive manner and has been useful in refining the target for conditions other than epilepsy.^7^, ^8^ In this preliminary work, we use tractography to evaluate the clinical utility of the connectivity between the SOZ and the whole thalamus. We hypothesize that clinical response is associated with the stimulation of the tracts connecting the SOZ with the thalamus in a patient‐specific manner.

## 2 MATERIALS AND METHODS

**FIGURE 1:**
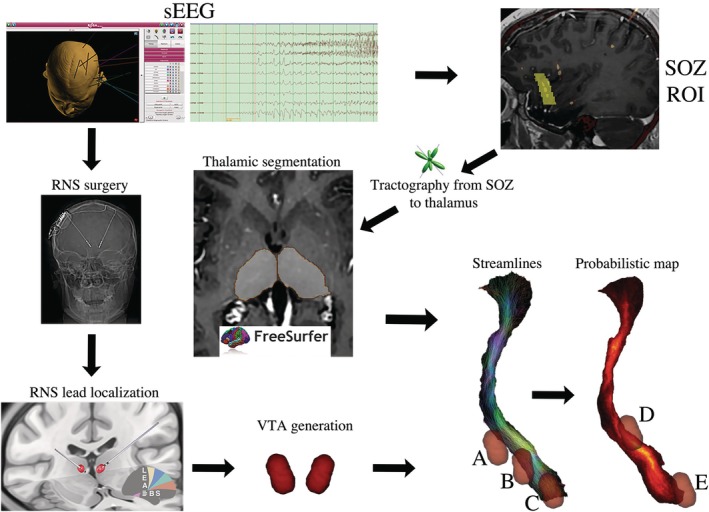
Workflow of the study. After stereoencephalography (sEEG), the seizure onset zone (SOZ) was manually drawn based on the contacts involved. Tractography was performed defining the SOZ as seeds and the patient‐specific thalamic segmentation as inclusion/waypoint region. Streamlines and probabilistic maps were obtained for further analyses. After sEEG, patients underwent thalamic responsive neurostimulation (RNS), and the electrodes were localized and the electrical field generated to determine the volume of tissue activation (VTA). Examples of the different proportions of the streamlines being included in each VTA (A, B, and C) were captured. Using the probabilistic maps, areas of high (D) or low (E) probability of connectivity between the SOZ and the thalamus being stimulated were captured as the highest probability peak within the VTA. ROI, region of interest.

This is a retrospective analysis of patients implanted with thalamic RNS between 2015 and 2023 at Mount Sinai Medical Center and University of Nebraska Medical Center. Institutional review boards of both institutions approved the study, and the patients consented to the use of their data in this study. These patients underwent intracranial stereoencephalography (sEEG) aiming to determine the SOZ and/or seizure network. SOZ is defined as the brain region where seizures originate. Only one patient was implanted with a single CM thalamic target with a depth electrode during sEEG. Visual review of sEEG is considered the gold standard to identify the SOZ in the current literature.^9^ In this study, a board‐certified epileptologist reviewed the sEEG in its entirety. The electrodes that were involved in the beginning of the seizures as determined by the epileptologist were considered SOZ electrodes. After the sEEG study, patients were discussed in multidisciplinary conference, and the decision was made to perform RNS with at least one thalamic target (Figure 1). The rationale to decide which thalamic nuclei to target was based on the idea that ANT is part of the Papez circuit and therefore better suited for seizures coming from temporal mesial structures and limbic areas.^10^ Centromedian nucleus, on the other hand, may be better for seizures coming from frontal, cingulate, and sensorimotor areas due to its extensive connections with these cortical areas.^2^

Baseline demographic data, seizure type/semiology, surgical history, and electrographic data were extracted from neurology and neurosurgery clinic reports available through the electronic medical record. Additional data on seizure frequency and semiology were gathered through structured interviews conducted with patients or their primary caregivers. Patients were asked to report the number of seizures experienced per month prior to RNS implantation (preoperative seizure frequency) and at the most recent follow‐up (postoperative seizure frequency) as of the time of the interview, at least 12 months postoperatively (Table 1). For cases where patients reported seizure frequency on a daily or weekly basis, the reported values were converted to a monthly frequency value after corroboration with the patient and cross‐referencing with NeuroPace device logs. If patients were unable to recall specific data during the interview, related data were retrieved from clinic notes documented by the treating neurologist or neurosurgeon. Seizure outcomes were also evaluated according to the Engel classification system, as determined by the attending neurologist or neurosurgeon at the time of follow‐up. To ensure reliable data collection, the date of epilepsy diagnosis, seizure semiology, surgical history, and demographic details were also reviewed and confirmed during the interview process.

**TABLE 1:** Clinical and demographic variables.

Case	Age, years/Gender	Seizure type	Duration of the epilepsy, years	Postoperative follow‐up, months	Prior resection, yes/no	RNS targets/Lat	Cortical target	Engel outcome
1	35/F	GTC	25	79	No	ANT/R	Yes, R SMA	IA
2	48/M	GTC	24	43	No	ANT/B	No	IA
3	18/F	Absence, FTBTC	13	38	No	ANT/B	No	IIB
4	34/F	FIA, FTBTC	14	31	No	CM/B	No	IVA
5	20/M	Absence, FTBTC	16.75	38	Yes	CM/B	No	IVC
6	16/M	FTBTC	10	35	No	ANT/B	No	IVA
7	22/M	FIA	6	29	No	CM/B	No	IIIA
8	18/M	GTC	3.83	67	No	CM/B	No	IIIA
9	25/M	FTBTC	8	39	No	CM/B	No	IA
10	22/F	FTBTC	11	31	No	CM/B	No	IIB
11	31/F	GTC	28	16	No	CM/B	No	IIIA
12	28/F	FTBTC	7	20	No	ANT/L	Yes, L hippocampus	IVB

Abbreviations: ANT, anterior nucleus of the thalamus; B, bilateral; CM, centromedian nucleus of the thalamus; F, female; FIA, focal impaired awareness; FTBTC, focal to bilateral tonic–clonic; GTC, generalized tonic–clonic; L, left; Lat, laterality; M, male; R, right; RNS, responsive neurostimulation; SMA, supplementary motor cortex.

### 2.1 RNS surgical technique and postoperative follow‐up

**FIGURE 2:**
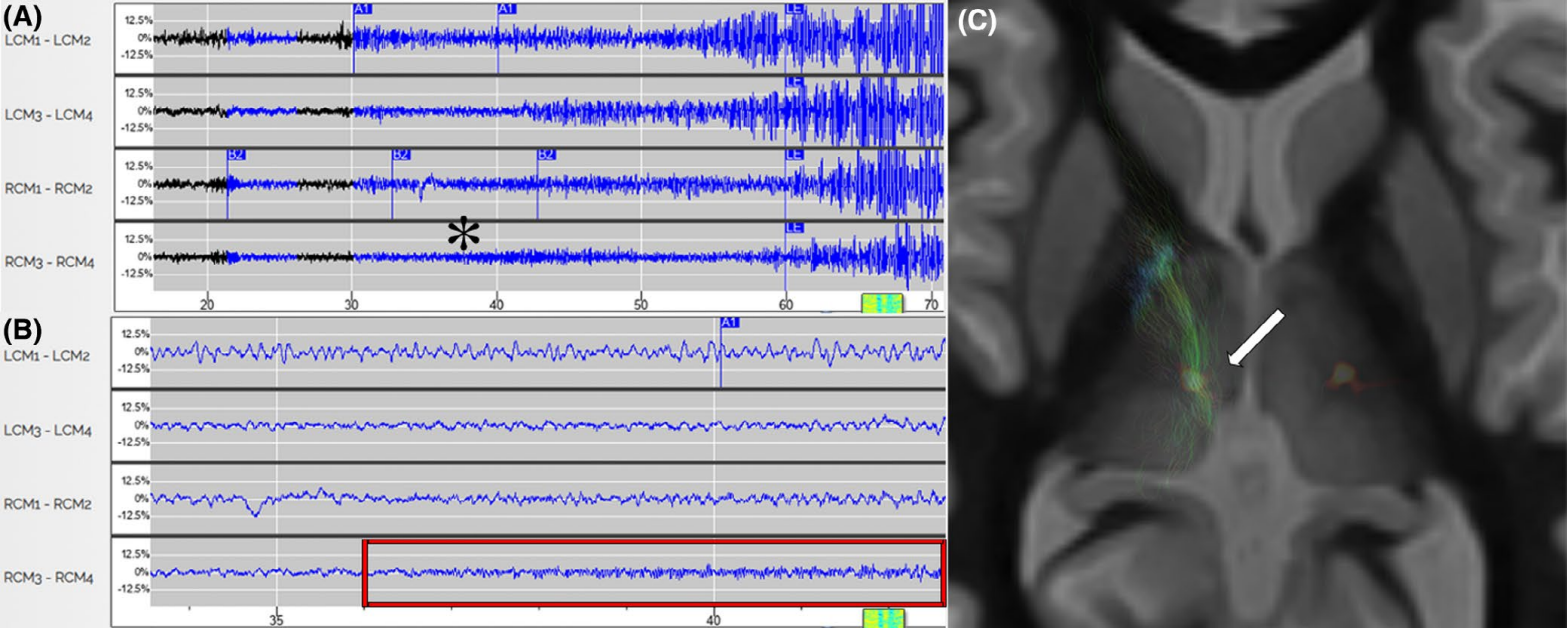
Thalamic seizure detections from the responsive neurostimulation electrocorticographic (ECoG) information. The y‐axis shows the channels (e.g., LCM_1_–LCM_2_ would be the first channel using the two distal contacts in the left centromedian nucleus; RCM, right centromedian nucleus), and the x‐axis shows the time in seconds. (A) Patient 3, long episode in the 70‐s ECoG shows ictal pattern (high frequency, 25 Hz) in the right centromedian nucleus, most robust in channel 4 (three and four contacts) seen at ~37 s (asterisk), before widespread high‐amplitude propagation across all channels. (B) Zoomed ECoG on the 10‐s view (red box) showing the high‐frequency ictal pattern on channel 4. (C) Axial view of the contacts where the ictal pattern was detected with the tracts connecting the seizure onset zone with the thalamus (arrow).

Thalamic targeting was performed using 3‐T fast gray matter acquisition T1 inversion recovery (FGATIR) and T1‐volumetric mapping with contrast in ROSA planning software (Zimmer Biomet). Both CM and ANT were implanted using direct targeting based on FGATIR MRI visualization. The ANT and the mammillothalamic tract (MMT) were clearly visible, and the anchor point of the target was selected based on the MMT–ANT junction using a transfrontal approach. MMT–ANT junction has been described before as a sweet spot for clinical effectiveness.^11^ For CM, the target was defined by direct visualization of the FGATIR sequence as the hypointense area ~3 mm lateral to the wall of the ventricle ~1 mm anterior and superior to the posterior commissure. Surgery was performed with patients under general anesthesia with robot‐assisted frameless stereotaxy using five bone fiducials for registration. An intraoperative computed tomography (CT) scan was acquired (Medtronic) and coregistered with preoperative magnetic resonance images. Head fixation was performed using a Leksell headframe (Elekta). Fiducial registration was performed and error < 1 mm was accepted in all cases. Depth electrodes in the thalamus (3.5‐mm spacing) and additional depths or strips at other targets were implanted according to the multidisciplinary conference plan. Intraoperative CT scan was performed to confirm accurate placement. Placement of the ferrule and implantable pulse generator replacements was performed in the parietal area followed by intraoperative electrocorticography. Four detection channels were defined with the two distal contacts of the left‐sided electrode as channel 1, two proximal contacts as channel 2, two distal contacts of the right‐sided electrode as channel 3, and two proximal contacts as channel 4. Standardized detections were set to estimate the morphology of the seizure onset and later refined based on individualized EEG data from captured seizure events (Figure 2). Postoperatively, we requested the patients to swipe the magnet when they felt a seizure, and this was stored for review. For the two patients with a cortical lead and a thalamic lead, detection was often on the cortical electrode alone with stimulation in both targets. Once the seizure was captured, detection was refined with the aim of being within 1 s of the onset. Stimulation was then started, typically with bipolar configuration activating all contacts. If the trajectory included contacts in the sensory thalamus, those contacts needed to be excluded to avoid facial paresthesias. We began with a stimulation intensity of .5 μC/cm^2^, 125 Hz, and duration of 200–5000 ms and titrated up at subsequent visits.

### 2.2 Imaging analyses

The preoperative T1‐weighted images were coregistered to the post‐sEEG CT scanner using advanced normalization tools in Lead‐DBS.^12^ We then preprocessed the 3‐T diffusion‐weighted imaging (DWI) with 65 gradient directions individually acquired in each patient preoperatively. We used FSL tools (FMRIB Software Library) to correct for subject motion, eddy currents, and susceptibility‐induced distortions.^13^ The corrected DWI was then coregistered to the T1‐weighted image that was previously coregistered to the post‐sEEG CT scan so that all images for analyses were in each patient‐specific space (native space). Fiber orientation distributions (FODs) were calculated in every voxel of the DWI coregistered to the anatomical T1 scan using the MRtrix software package (Brain Research Institute).^14^ After the SOZ was defined by the epileptologists, anatomical areas within the SOZs were defined in the T1 volumetric scan coregistered with post‐sEEG CT scan. Using MRview, we manually segmented a region of interest (ROI) around each specific SOZ electrode contact (sphere of ~10‐mm radius) to encompass both the cortical area being sampled and its adjacent white matter (WM) to obtain an anatomically meaningful segmentation.^15^ Thalamic segmentation was applied using a FreeSurfer algorithm for patient‐specific thalamic segmentation.^16^ This algorithm allows thalamic segmentation into 25 different nuclei, using a probabilistic atlas built with histological data using the patient‐specific T1‐weighted image. We were interested in using the segmentation of the whole thalamus to set it as inclusion/waypoint region for the tractography analysis. The whole tractography analysis was performed in each patient native space individually. To perform the fiber tracking between the SOZ and the patient‐specific thalamic segmentation, we used a constrained spherical deconvolution (CSD) probabilistic tractography algorithm defining 5000 streamlines with an FOD amplitude cutoff of .1. The seed region was defined as the SOZs obtained by manual segmentation as described above and the patient‐specific thalamic segmentation as inclusion/waypoint region. In cases where the streamlines passed adjacent to the thalamus and terminated in the brainstem, these streamlines were excluded from the analysis to only have WM trajectories connecting to the thalamus. In case of two or more SOZs unilateral or bilateral, the tract between each SOZ and the thalamus was obtained separately and then these multiple SOZ tracts were added into a single tract per patient for the correlation analysis. The *streamlines* represent the trajectories of the real WM fibers connecting the SOZ to the thalamus. Even though the number of streamlines was set at 5000 as described above, the final number of streamlines was variable depending on the number of SOZs in each patient and the streamlines that only reached the thalamus based on the patient‐specific diffusion signal. Then, to obtain the probability of connectivity along the tract connecting the SOZs with the thalamus, these tracts were converted into high‐resolution *probabilistic maps* with the standard thresholding at 95% probability of connectivity (Table 2).

**TABLE 2:** Imaging analysis data.

Case	SOZ brain location	SOZ ROI volumes, mm^3^	Multiple SOZ <> thalamus pattern of connectivity	Stimulation parameter contacts, mA	VTA volume per side, mm^3^	SOZ streamlines reaching the thalamus, *n*	SOZ streamlines activated, *n*/%	Seizure frequency, preop, average per month	Seizure frequency, postop, average per month	Seizure frequency reduction, %
1	R SMA	3617, 4766	—	R: 0+−0 .5	88R	10 000	2883/29	.17	0	100
2	R aCing	1360	—	L: +−00 2 R: +−00 2	127L/128R	5000	798/16	.33	0	100
3	R OFC, R pIns, R mesT	7673, 8027, 2477	Transthalamic	L: 0+−0 2.9 R: +−00 2.9	248L/237R	13 237	2015/15	750	4	95
4	R OFC, R aIns	1302, 2188	Convergent	L: +−+− 3 R: +−+− 3	247L/245R	8846	447/5	4	3	25
5	L&R pCing, L&R pIns	9360, 3751, 2427, 3658, 4158	Convergent	L: +−+− 4 R: +−+− 4	337L/340R	25 000	1174/5	2	4	0
6	R OFC, R mesT	2391, 2869	Transthalamic	L: +−+− 4 R: +−+− 4	332L/339R	10 000	926/9	12	10	16.67
7	R SMA, R aCing	3344, 2084	Convergent	L: +−+− 6 R: +−+− 6	745L/746R	10 000	1936/19	2	.8	60
8	L&R mPFC	4410, 4094	—	L: +−+− 4 R: +−+− 4	285L/282R	10 000	1020/10	900	450	50
9	L&R aCing, L pIns	3166, 3216, 3428	Convergent	L: +−00 1.5 R: 00+− 1.5	146L/100R	5150	805/16	3	0	100
10	R mPFC, R SMA	4022, 2760	Convergent	L: +−00 3 R: 00+− 3	217L/250R	4721	1066/23	9	.33	96.33
11	L OFC, L aIns	1300, 2059	Convergent	L: +−+− 2.5 R: +−+− 2.5	227L/229R	10 000	2557/26	4	1	75
12	L mesT	1388, 1685, 1343	—	L: +−+− 2	199L	15 000	862/6	5	6	0

*Note*: The number of streamlines reaching the thalamus derived from the individual SOZ ROIs was set by default at 5000. The final number of streamlines reaching the thalamus depended on the individual diffusion signal and the number of SOZ ROIs individually processed for each patient. On the other hand, the number of streamlines activated by stimulation was derived from the subtraction of the streamlines passing through the VTA calculated as a proportion of the whole SOZ tract rather than the absolute number. Seizure frequency preop and postop was calculated as percentage of seizure reduction.

Abbreviations: aCing, anterior cingulate cortex; aIns, anterior and posterior insular cortex; L, left; mesT, mesial temporal cortex; mPFC, medial prefrontal cortex; OFC, orbitofrontal cortex; pCing, posterior cingulate cortex; pIns, posterior insular cortex; postop, postoperatively; preop, preoperatively; R, right; ROI, region of interest; SMA, supplementary motor cortex; SOZ, seizure onset zone; VTA, volume of tissue activation.

To calculate the *volume of tissue activation* (VTA), we used the individual electrode contact locations, regardless of the intended target. The electrodes were localized using Lead‐DBS, and stimulation parameters at last follow‐up were used to calculate the VTA as described elsewhere (Figure 1).^17^ In short, a four‐compartment model of the area around the electrode is constructed based on the electrode itself (insulating and conducting parts) and the gray matter and WM of the brain. The regions within this compartment were defined as follows. The subcortical gray matter structures were defined by the DISTAL atlas, the electrode's parts were defined by the virtual electrode models provided by the software, and the regions not covered by electrode or gray matter were modeled as isotropic WM. The anisotropic conductivity values for gray matter and WM were defined as *σ* = .33 and .14 S/mm respectively. The electric field threshold was set to *e* = .2 V/mm, and the resultant binary VTA was obtained thresholding the electrical field strength at the specific amplitude and pulse‐width used for each stimulation setting.

Finally, given the potential changes in connectivity related to epilepsy, we compared the structural connectivity between the SOZ and the thalamus of our patients with a group of healthy subjects. We used a high angular resolution DWI human template of 72 healthy subjects in the Montreal Neurological Institute (MNI) standard space, freely available online (https://www.nitrc.org/projects/iit/). FODs were estimated with CSD probabilistic tractography using the same default parameters mentioned above on MRtrix. We nonlinearly coregistered the T1‐weighted images of our patients to the healthy subjects DWI template in MNI standard space using FSL tools. This allowed the transformation of the SOZ ROIs from patients native space to standard MNI space. To maintain consistency in the thalamic anatomy used for the patient analysis, we applied the thalamus segmentation FreeSurfer algorithm on the T1‐weighted image derived from the same 72 healthy subjects template. We performed tractography from each SOZ transformed into the standard MNI space to the thalamus using the same parameters mentioned above.

### 2.3 Statistical analyses

We initially categorized the patients into responders (>50% reduction in seizure frequency), partial responders (50%–25%), and nonresponders (<25%) based on the seizure frequency at baseline compared to the last follow‐up. For the purposes of the first statistical analysis, the nonresponder (*n* = 3) and the partial responder (*n* = 2) groups were added into a single group (*n* = 5) to compare with the responders (*n* = 7). To define whether the VTA was activating low‐ or high‐probability areas from the tracts connecting the SOZ with the whole thalamus, we captured the highest probability peak within the VTA in each patient that was obtained based on individual electrode locations, regardless of the intended target nuclei. We compared the median probability of connectivity of the tract being activated by therapeutic stimulation between response groups (responders vs. partial/nonresponders) using the nonparametric Mann–Whitney *U*‐test. For the second analysis, the seizure frequency outcomes and the amount of SOZ tracts activated by therapeutic stimulation were treated as continuous variables. To define the proportion of stimulated tracts from the SOZ to the thalamus, we captured the proportion of streamlines included in the VTA from the whole tract. If patients had bilateral thalamic stimulation or multiple/bilateral SOZs, the unilateral or bilateral activation of these multiple tracts were added for each patient to produce a single number per patient for the correlation analysis. We then performed a correlation analysis between the total proportion of the streamlines being activated by therapeutic stimulation and the seizure reduction at last follow‐up using nonparametric Spearman rank correlation coefficient (two‐tailed). For both statistical analyses, significance level was set at <.05 and the GraphPad Prism software package was used for computations.

## 3 RESULTS

### 3.1 Clinical

**FIGURE 3:**
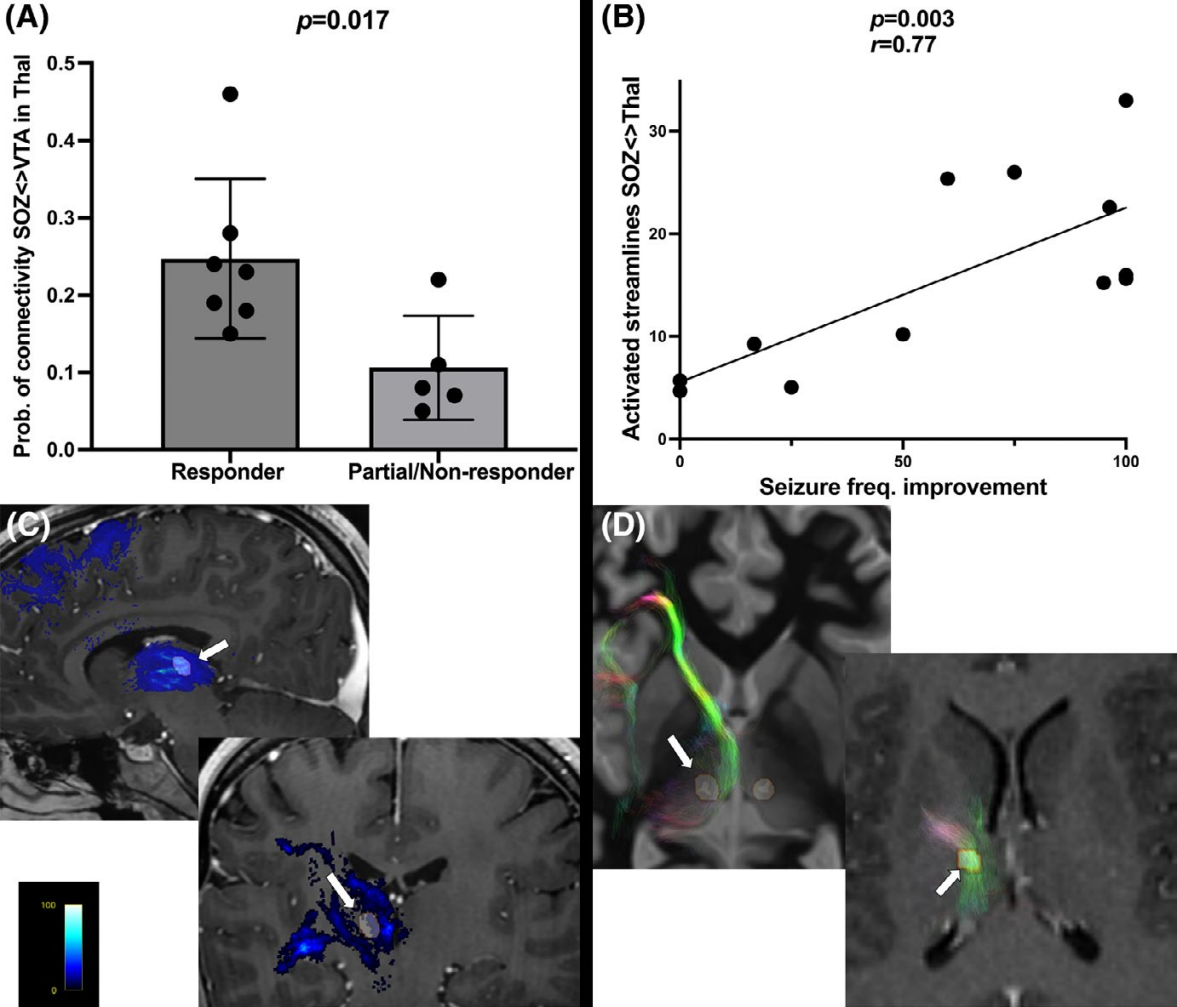
(A) Responders activated significantly higher probability of connectivity areas between the seizure onset zone (SOZ) and the thalamus when compared with partial/nonresponders. (B) The degree of tract activation by therapeutic stimulation positively correlated with seizure reduction at last follow‐up. (C) Upper panel: Patient 10 (responder) with therapeutic stimulation activating higher probability of connectivity areas between the SOZ and the thalamus (arrow). Lower panel: Patient 4, partial responder with the volume of tissue activation (VTA) located mostly in low probability of connectivity areas between the SOZ and the thalamus (arrow). (D) Upper panel: Patient 4 (partial responder) with VTA missing the main bundle of SOZ tracts connecting with the mediodorsal nucleus (arrow). (D) Lower panel: Patient 1 (responder) with VTA located in the main bundle of SOZs tracts connecting with the thalamus (arrow).

Twelve patients were included in this study (six male, six female) with a mean age of 26 years and mean follow‐up of 38 months. Clinical, demographic, and seizure outcome variables are recorded in Table 1. Thalamic nuclei targeted included ANT (*n* = 5) and CM (*n* = 7). Ten patients were treated with bilateral thalamic stimulation and two patients with unilateral thalamocortical stimulation (Table 1). Thalamic stimulation was delivered using quadripolar configuration in seven patients and bipolar configuration in five patients. Seven patients were responders, two partial responders, and three nonresponders at last follow‐up. The connectivity analysis showed that the median probability of connectivity between the SOZ and the thalamus was significantly higher within the stimulated area of responders (median = .23, interquartile range [IQR] = .18–.28) compared with partial/nonresponders (median = .08, IQR = .06–.17; *p* = .017). This result suggests that stimulation of thalamic areas most likely connected with the SOZ may be associated with clinical response. On the other hand, the proportion of the tracts between the SOZ and the thalamus being activated by the stimulation was variable in between patients (Figure 1). There was a strong positive correlation between the proportion of activated SOZ‐to‐thalamus tracts and seizure reduction (*p* = .004, *r* = .78), suggesting that a higher proportion of stimulated tracts from SOZ to thalamus may correlate with a greater therapeutic effect on seizure reduction (Figure 3).

### 3.2 Anatomical

**FIGURE 4:**
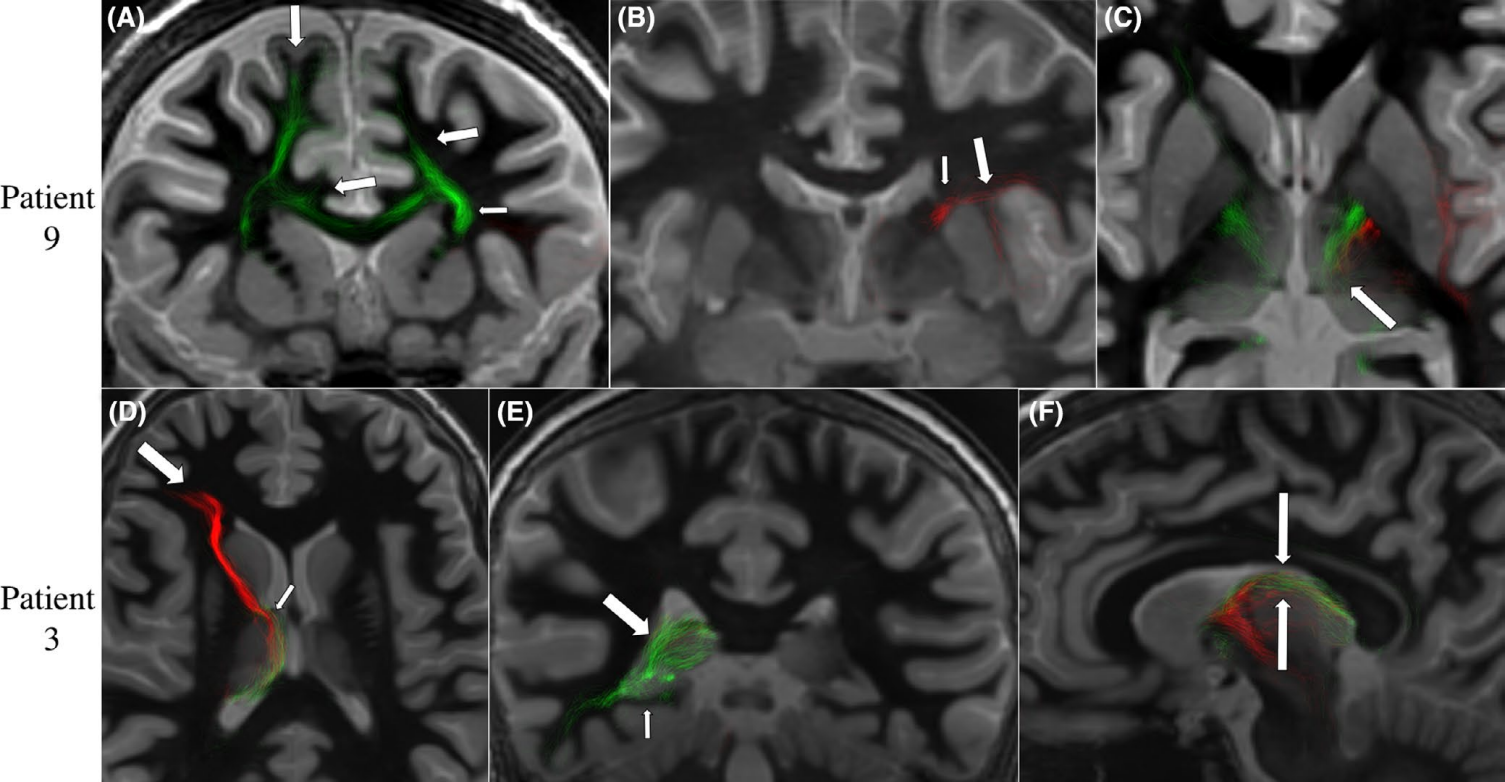
Patterns of connectional anatomy of the seizure onset zone (SOZ) with the thalamus: case examples. Patient 9 had multiple SOZs in supplementary motor (SMA), anterior cingulate (aCing), and posterior insular (pIns) cortex. (A) SMA and aCing SOZ tracts (green, large arrows) reached the thalamus through the internal capsule (small arrow). (B) pIns SOZ tracts (red, large arrow) also reached the thalamus through the internal capsule (small arrow). (C) All SOZs tracts converged in the CM (arrow). For Patient 3, SOZs were in orbitofrontal cortex (OFC) and mesial temporal cortex. (D) Axial view showing that OFC SOZ tracts (red, large arrow) reached the thalamus through the internal capsule connecting with the anterior nucleus of the thalamus (ANT; small arrow). (E) Coronal view showing the posterior mesial temporal SOZ tracts (green) reaching the thalamus through the temporothalamic bundle and connecting with the pulvinar (Pulv; large arrow). (F) Sagittal view showing OFC SOZ connection with the ANT (red) and the mesial temporal (green) SOZ tracts reaching the posterior aspect of the ANT after passing through the Pulv following a transthalamic pattern between two anatomically distant SOZs.

**FIGURE 5:**
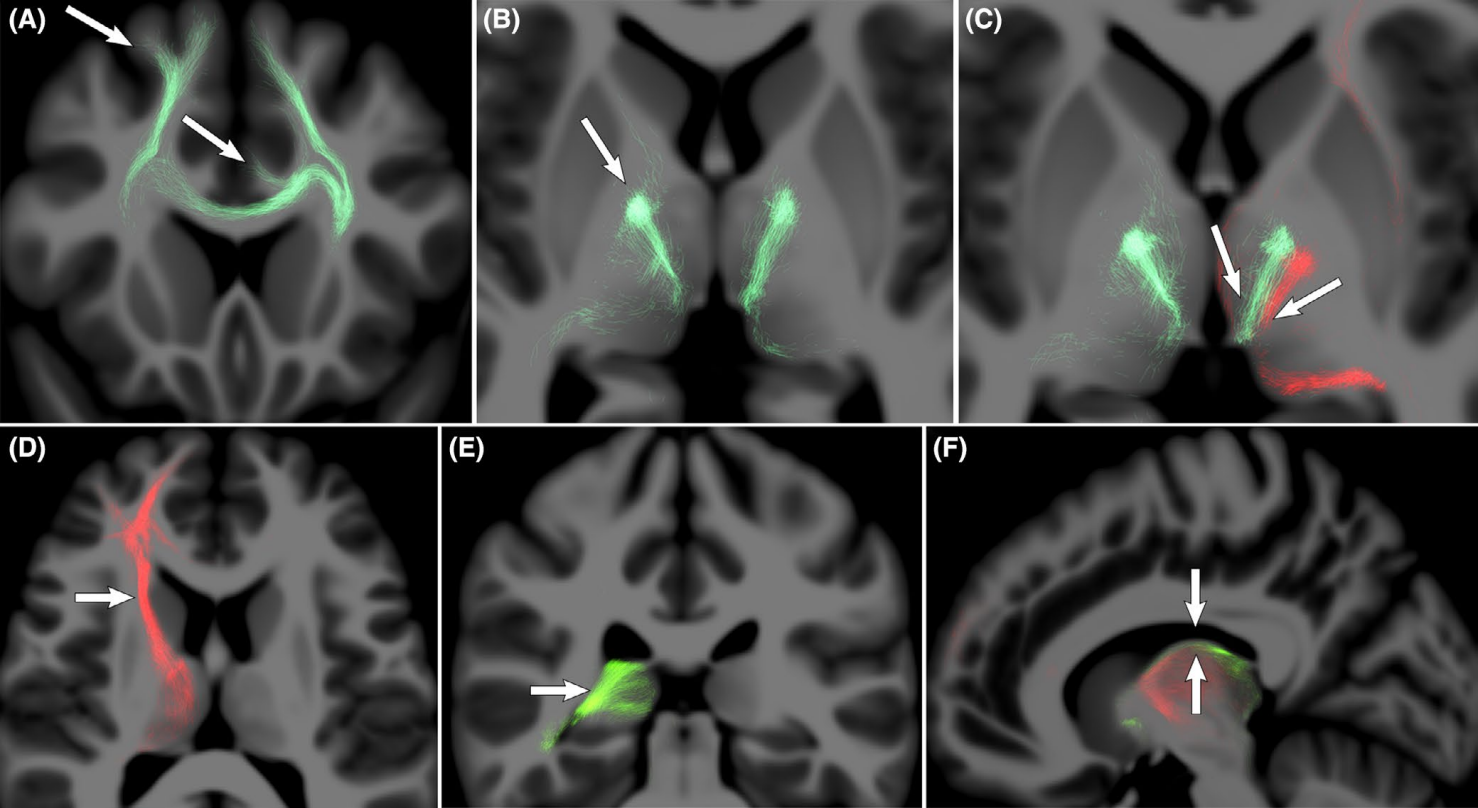
Tractography analysis of healthy subjects to compare the seizure onset zone (SOZ) connections found in our patients. SOZ regions of interest were transferred to the standard Montreal Neurological Institute space and analyzed in the diffusion‐weighted imaging template of the healthy subjects. Frontal, insular, and cingulate areas connected through the internal capsule with the centromedian nucleus (A–C) of the thalamus showing a convergence pattern of connectivity (arrows in C). Orbitofrontal cortex also connected with the thalamus through the internal capsule (D), whereas temporal areas connected with the thalamus through the temporothalamic bundle (E), reaching both the pulvinar and anterior nucleus of the thalamus in a transthalamic manner (arrows in F).

The SOZs in our patients were located in medial prefrontal (mPFC), supplementary motor (SMA), anterior cingulate (aCing), posterior cingulate, orbitofrontal (OFC), anterior and posterior insular, and mesial and lateral temporal (mesT, latT) cortices. The connectional anatomy between these cortical areas and the thalamus demonstrated a complex pattern of connectivity showing a connectivity gradient along several thalamic nuclei. For instance, mPFC areas connected to different thalamic nuclei including ANT, MD, and CM. Also, SMA and aCing connected to the intralaminar nuclei including the CM. Mesial and lateral temporal areas connected with the posterior thalamus along different subdivisions of the Pulv, with some fibers reaching the posterior aspect of the ANT. Frontal, cingulate, and insular areas connected with the thalamus through the internal capsule and temporal areas through the temporothalamic bundle. Furthermore, connections of multiple SOZs and the thalamus showed two different anatomical patterns. The first anatomical pattern showed two or more relatively close SOZs connecting with the same thalamic nucleus through the same WM route; this was the *convergent* pattern. For example, SMA, insular, and aCing SOZs tracts connected with the thalamus through the internal capsule and converged into the CM. The second anatomical pattern showed two relatively distant SOZs connecting to each other following a “*transthalamic*” pattern through different thalamic nuclei and different WM routes. For example, OFC and mesial temporal SOZs connected to each other through the ANT. OFC, mPFC, and aCing SOZ tracts entered the dorsal internal capsule and connected with the ANT. On the other hand, mesT and latT SOZ tracts entered the thalamus through the temporothalamic bundle, and then through the Pulv some fibers connect to the posterior aspect of the ANT (Figure 4). The tractography analyses in the DWI of the healthy subjects revealed similar patterns of connectivity as found in our patients. These SOZ areas showed a gradient of connectivity along the thalamic nuclei (Figure 5).

## 4 DISCUSSION

Thalamic RNS is a surgical option for patients with DRE,^3^, ^4^ but individualized thalamic targets based on the patient‐specific seizure network are still unknown. Prior evidence suggests that the degree of connectivity between the stimulated targets and epileptogenic networks plays a crucial role in therapeutic efficacy.^18^ Therefore, targeting thalamic areas that are connected with the seizure network might represent a more clinically efficacious approach compared with the traditional nuclei‐based target selection. One of the most effective clinical tools to identify surgical targets is the definition of the seizure network during sEEG. However, given the size and complexity of the thalamus, there is still uncertainty on how to optimally include the thalamus during sEEG studies. On the other hand, tractography has been used for more than 2 decades to define brain circuits noninvasively and help in the identification of neuromodulation targets.^19^ This technique has successfully guided neurosurgical targeting in patients with brain disorders other than epilepsy such as tremor, Parkinson disease, depression, and obsessive–compulsive disorder.^8^, ^20^, ^21^ In this preliminary work, we included 12 patients with SOZs in different areas of the brain treated with thalamic RNS in two different nuclei of the thalamus (ANT and CM). We calculated the connectivity between the SOZs and the thalamus in a patient‐specific manner and investigated the relationship between the stimulation of these connections and clinical outcomes. We used patient‐specific high angular resolution CSD‐probabilistic tractography to identify connections between the SOZ and the thalamus. This connectivity analysis suggested that stimulating tracts with higher probability of connectivity between the SOZ and the thalamus may be associated with a clinical response of more than 50% seizure reduction, regardless of the intended target nucleus. Additionally, we found that the activation of these tracts by therapeutic stimulation may correlate with seizure reduction.

Thalamic RNS is increasingly being used for multiple SOZs or as a supplement of cortical targets. Recent advances in the understanding of the role of the thalamus in epilepsy make the thalamus a potential hub for stopping seizure activity if its connections with the seizure networks are considered.^22^ However, we are still using the same traditional thalamic DBS targeting principles for RNS, and the patient‐specific seizure network is not systematically taken into consideration. Given that epilepsy is a network disorder, we postulate that connectivity between SOZ and thalamus should be considered when selecting these thalamic targets. Recently, other thalamic nuclei have been identified as potential neuromodulation targets for epilepsy, including MD,^6^ Pulv,^5^ lateral geniculate,^23^ and ventral intermediate nucleus (Vim).^24^ However, these new targets have been selected based on anatomical assumptions and not on a personalized evaluation of patient‐specific seizure networks. Based on our analysis, stimulating the connections between the SOZ and the thalamus may be associated with clinical response and may correlate with seizure outcomes. Therefore, mapping the seizure network as it integrates with the thalamus could reveal areas to target that are more clinically effective and potentially more efficient than the traditionally targeted nuclei. Although not formally tested, in some cases we found that the specific contacts associated with clinical benefit were not located in the intended target but in the WM bundle connecting the SOZ with the thalamus. This may lead us to think that stimulating the intrathalamic WM tracts may have more impact on clinical outcomes than stimulating at the intended target nuclei. For instance, this has already been described in the literature on ANT DBS for DRE, where electrode contacts at the MMT seem to be more effective than the contacts within the nucleus.^11^ This has also been the case for patients with essential tremor treated with Vim DBS. Electrode contacts in the WM ventral to the Vim (dentatorubrothalamic tract) are more effective and efficient than in the Vim proper.^25^ These data are consistent with the idea that the clinical effect of neuromodulation is more driven by WM than gray matter stimulation, which has already been postulated in neuromodulation for epilepsy.^26^

To target the SOZ connections with the thalamus, it is necessary not only to identify the nodes but also the anatomy of the connections between the SOZ and the thalamus, the “thalamic seizure network.” Cortical areas harboring the SOZ connect with the thalamus through different WM routes, including the internal capsule and the temporothalamic bundle.^10^ Once in the thalamus, these SOZ connections displayed two main patterns of connectivity: convergent and transthalamic. The *convergent* pattern showed two or more relatively close SOZs connecting with the same thalamic nucleus through the same WM route. In the *transthalamic* pattern, one SOZ seems to connect with a different and relatively distant SOZ through the thalamus traversing different thalamic nuclei.^27^ This connectivity pattern seems to create a transthalamic corticocortical connection centered at a higher order thalamic nucleus. Convergent and transthalamic communication has been described at the microscale as a mechanism of corticothalamocortical communication.^28^, ^29^ Here, we described at a macroscale how the SOZs coming from different cortical areas connect with the thalamic nuclei. These two patterns of connections of the SOZ with thalamus may play a role in the integration of the SOZs into a more global seizure network.^30^ Some evidence suggests that the thalamus may act as the main hub for recurrent epileptogenic networks after surgical resection.^31^ Some evidence also suggests that higher order thalamic nuclei (i.e., ANT, Pulv, CM, and MD) may facilitate corticocortical transthalamic communication by synchronizing oscillatory activity, and this mechanism may play a role in seizure generalization.^32^

Including the thalamus during sEEG studies is another strategy to maximize the target selection in the thalamus. Recent evidence shows that including the thalamus in sEEG is safe and effective in capturing ictal activity.^33^ Insights from these studies are already revealing other potential thalamic targets.^33^, ^34^ One such study found that the pulvinar captured earlier seizure activity from the temporal lobe compared with the ANT, which is a more established target for temporal lobe epilepsy.^33^, ^35^ This isolated evidence reinforces the idea that the current selection of thalamic targets is suboptimal and including the thalamus during sEEG may be useful in refining thalamic target selection. However, the thalamus is composed of ~40 nuclei clustered in different areas with different anatomical configurations. The coverage of these nuclei would be challenging without a clinical hypothesis for implantation. To develop clinical hypotheses based on noninvasive phase I studies, connectivity information between sampled cortical areas and the thalamus may be considered. However, the corticothalamic connectivity is not merely limited to discrete thalamic nuclei connected with discrete cortical areas but depicts a more anatomically complex set of intrathalamic WM paths creating a gradient of connectivity along the thalamic nuclei.^19^, ^36^ Finding the most efficient spot along this gradient using tractography may be the key to improving outcomes in thalamic neuromodulation for epilepsy.

This work has several limitations, including the small number of subjects and the retrospective nature of the study. Prospective studies with larger numbers of patients are needed to confirm these findings. Moreover, we included patients with SOZs located in different brain regions, including frontal, cingulate, insular, and temporal. These patients underwent unilateral and bilateral stimulation of two different thalamic nuclei (ANT and CM). Therefore, the relationship between connectivity and clinical outcomes is only suggestive. However, even though the SOZs were in different areas of the brain, the primary endpoint of the study was the connectivity between these areas and the thalamus regardless of its specific location in the cortex. The same consideration applies to the thalamic targeting; even though the intended targets were CM and ANT, the analysis was based on the electrical field around the individual contact positions as they are spatially related to the SOZ tracts regardless of the intended target. This allowed us to do a more patient‐specific assessment of the seizure network engagement by the therapeutic stimulation. Furthermore, thalamocortical stimulation could have potentially biased our results, as two patients had thalamic and cortical RNS, and 10 patients had bilateral thalamic stimulation. From these patients, one was a responder and the other was a nonresponder; this suggests that combined thalamocortical stimulation may not have significantly impacted our results.

Tractography analyses have well‐known limitations that have been described elsewhere.^37^ Here, we used CSD‐probabilistic and patient‐specific tractography for individualized connectivity analyses, and we used advanced coregistration tools to obtain an accurate coregistration in the setting of brain structural abnormalities that are relatively frequent in epilepsy patients. Manual segmentation of the SOZ has limitations due to the lack of established knowledge of the radius of recording of an sEEG electrode contact. In a previous paper, the segmentation of a sphere around the contact involved in the seizure onset was used to get an anatomically meaningful area of the SOZ.^15^ In this work, we aimed to create anatomical ROIs including cortex but also WM to increase the likelihood of finding projection pathways connecting with the thalamus. Tractography analyses using only cortical areas (without adjacent WM) will reduce the sensitivity to find projection pathways and will mostly find short association pathways. Electrical field analyses also have well‐known limitations related to the methodology that have been described elsewhere.^38^ More complex electrical field analyses have been deemed methodically superior to the VTA model.^39^ However, the VTA has been the only model that has demonstrated clinical utility in different targets including the thalamus in a real‐world setting.^40^

Another limitation is the lack of electrophysiological validation of this SOZ to thalamic connectivity. Future studies involving patients with thalamic sEEG electrodes could demonstrate seizure spread into thalamic areas with higher or lower probability of connectivity with the SOZ. This could provide markers of electrophysiological connectivity that could validate the WM connectivity and correlate with the epileptogenicity of the different SOZs.^41^ Single pulse stimulation could be another strategy to validate the structural connectivity between the thalamus and the SOZ areas.^42^ The sEEG‐derived SOZ is limited in that seizure detection can only be delineated from the implanted electrodes, which are based on the clinical hypotheses. Therefore, the connectivity analyses are limited to the areas hypothesized and sampled, which narrows the potential for a more complex picture of the corticothalamocortical anatomy. Aggregated data from multiple patients can be useful to identify the full anatomy of these networks and their clinical relevance.

## 5 CONCLUSIONS

It is well known that identifying and disrupting the seizure network is associated with optimal outcomes after surgical treatment of DRE. The thalamus has gained interest as a target for neuromodulation in epilepsy, but thalamic areas connected to the patient‐specific epilepsy network have not been identified as potential surgical targets with impact in clinical outcomes. Our results suggest that stimulation of the WM connections between the SOZ and the thalamus is associated and may correlate with better seizure outcomes. Understanding the patient‐specific “thalamic seizure network” and the surgical targeting of these connections may result in improved clinical outcomes in patients treated with thalamic RNS.

## AUTHOR CONTRIBUTIONS

**Josue M. Avecillas‐Chasin and Juan A. Barcia:** Conceptualization; design; methodology; execution of the imaging analysis. **Saadi Ghatan, Lara Marcuse, Madeleine Fields, Maite La Vega‐Talbott, Hesham T. Ghonim, Lakshman Arcot Jayagopal, Varun R. Subramaniam, Andy Ho Wing Chan, Daniel D. Cummins, and Yunju Im:** Acquisition and analysis of clinical data. **Fedor Panov, Josue M. Avecillas‐Chasin, and Saadi Ghatan:** Supervision, data curation of the whole study. **Josue M. Avecillas‐Chasin:** Drafting initial version of the manuscript. All authors review, edited, and approved the final version of the manuscript.

## FUNDING INFORMATION

The project described is supported by the National Institute of General Medical Sciences, U54 GM115458, which funds the Great Plains IDeA‐CTR Network. The content is solely the responsibility of the authors and does not necessarily represent the official views of the NIH.

## CONFLICT OF INTEREST STATEMENT

None of the authors has any relevant conflict of interest to disclose. We confirm that we have read the Journal's position on issues involved in ethical publication and affirm that this report is consistent with those guidelines.

## ETHICS STATEMENT

The study protocol was approved by the institutional review boards for research with human subjects at Mount Sinai Hospital and University of Nebraska Medical Center.

## PATIENT CONSENT STATEMENT

All participants signed consent for analysis of the data.

## Data Availability

Data supporting these findings are available from the corresponding author upon reasonable request.
